# Early myocardial damage (EMD) and valvular dysfunction after femur fracture in pigs

**DOI:** 10.1038/s41598-021-86151-z

**Published:** 2021-04-19

**Authors:** Birte Weber, Ina Lackner, Theodore Miclau, Jonathan Stulz, Florian Gebhard, Roman Pfeifer, Paolo Cinelli, Sascha Halvachizadeh, Michel Teuben, Hans-Christoph Pape, Miriam Lipiski, Nikola Cesarovic, Miriam Kalbitz

**Affiliations:** 1 Department of Traumatology, Hand, Plastic and Reconstructive Surgery, Center of Surgery, University of Ulm, Albert-Einstein-Allee 23, 89081 Ulm, Germany; 2 Department of Orthopaedic Surgery, Orthopaedic Trauma Institute, University of California, San Francisco, USA; 3 Department of Trauma, University Hospital of Zurich, Zurich, Switzerland; 4 Department of Surgical Research, University Hospital of Zurich, Zurich, Switzerland; 5 Department of Health Sciences, Translational Cardiovascular Technologies, Swiss Federal Institute of Technology, Zurich, Switzerland

**Keywords:** Cardiology, Medical research

## Abstract

Musculoskeletal injuries are the most common reason for surgery in severely injured patients. In addition to direct cardiac damage after physical trauma, there is rising evidence that trauma induces secondary cardiac structural and functional damage. Previous research associates hip fractures with the appearance of coronary heart disease: As 25% of elderly patients developed a major adverse cardiac event after hip fracture. 20 male pigs underwent femur fracture with operative stabilization via nailing (unreamed, reamed, RIA I and a new RIA II; each group n = 5). Blood samples were collected 6 h after trauma and the concentration of troponin I and heart-type fatty acid binding protein (HFABP) as biomarkers for EMD were measured. At baseline and 6 h after trauma, transesophageal ECHO (TOE) was performed; and invasive arterial and left ventricular blood pressure were measured to evaluate the cardiac function after femur fracture. A systemic elevation of troponin I and HFABP indicate an early myocardial damage after femur fracture in pigs. Furthermore, various changes in systolic (ejection fraction and cardiac output) and diastolic (left ventricular end-diastolic pressure, mitral valve deceleration time and E/A ratio) parameters illustrate the functional impairment of the heart. These findings were accompanied by the development of valvular dysfunction (pulmonary and tricuspid valve). To the best of our knowledge, we described for the first time the development of functional impairment of the heart in the context of EMD after long bone fracture in pigs. Next to troponin and HFABP elevation, alterations in the systolic and diastolic function occurred and were accompanied by pulmonary and tricuspid valvular insufficiency. Regarding EMD, none of the fracture stabilization techniques (unreamed nailing, reaming, RIA I and RIA II) was superior.

## Introduction

Musculoskeletal injuries are the most common reasons for surgery in severely injured patients and 70% of patients need an orthopedic surgery after trauma^1^. Femur fractures represent a significant public health problem in particular in the elderly population with an 1-year mortality up to 27%^2^. In addition to direct cardiac damage after physical trauma, there is rising evidence of trauma-induced secondary cardiac injury (TISCI)^3–5^. In different long bone fracture models, a present study describes the role of tumor necrosis factor; complement factor 5a and extracellular histones in mediating cardiac alterations after trauma^6^. Whereas, those mediator are described to mediate cardiac dysfunction with a reduction of the ejection fraction and shortening faction in multiple injured pigs^7^, the exact interplay between long bone injuries, therapy and cardiac function however, remains unclear.

Long bone fractures were described to consolidate faster and more reliably if stabilization with an intramedullary nail was conducted in combination with reaming of the intramedullary canal^8^. Nevertheless, reaming was associated with the occurrence of fat embolism, which might have crucial consequences for the lung^9^. During intraoperative echocardiography, 8 out of 20 patients showed embolic particles upon intramedullary reaming while no embolic events were configurated using an unreamed technique^9^. Nevertheless, more recent studies showed less embolism after reaming compared to unreamed nailing in the experimental setting of femur shaft fracture in sheep^10^ and no pulmonary functional differences between reamed and unreamed nailing in pigs^11^. Next to risking respiratory consequences due to embolism, the heat of the reamer is discussed to affect the intraosseous blood supply via necrosis^12^. In order to overcome these issues, the Reaming-Irrigator-Aspirator (RIA) was developed, which works with a head cooled by fluid and a negative pressure suction that simultaneously cleans the intramedullary canal from reaming debris^13^. Despite these advantages, the systemic increase of inflammatory mediators after reaming remains controversial. Some authors acknowledge the differences between reaming and unreamed nailing in the systemic concentrations of inflammatory mediators like IL-6^14–16^. Following this research, the present study analyzes the differences in operative techniques—unreamed nailing, reaming, RIA I and a novel RIA II system—with regard to early myocardial (EMD) damage after trauma.

We hypothesized that femur fracture in pigs leads to EMD with a troponin elevation, cardiac dysfunction and impaired valvular function. Additionally, we expected cardiac alterations in structural proteins as well as cell-to-cell contacts.

## Results

**Figure 1:**
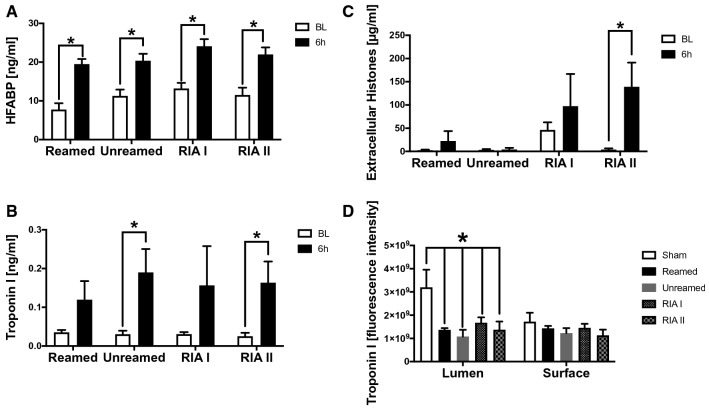
Myocardial Damage: (**A**) Systemic Heart-Fatty Acid Binding Protein (HFABP) in ng/ml in serum samples of pigs at Baseline (white) and 6 h after femur fracture (black) and either nailing (unreamed), reaming (reamed), Reamer-Irrigator-Aspirator 1 (RIA1) or RIA 2. (**B**) Systemic Troponin I measured in ng/ml in serum samples. (**C**) Systemic elevation of extracellular histones in µg/ml serum bevor and after femur fracture. (**D**) Quantitative analysis of troponin stained section of the left ventricle 6 h after femur fracture. n = 5 in each group, data were presented as ± SEM, *p ≤ 0.05.

In order to determine the occurrence of EMD, the classical cardiac damage marker troponin I was analysed at baseline and 6 h after fracture. We observed an increase of systemic troponin I after femur fracture and nailing, as well as after RIA II and subsequently nailing (Fig. 1B). Furthermore, troponin I staining of the left ventricle showed a significant decrease of local troponin I concentration in the luminal layers of all treated animals, compared to sham animals (Fig. 1D). This might be explained by the systemic increase of troponin. Because of the early timepoint after trauma, we additionally measured the HFABP and detected a systemic increase in all included animals 6 h after fracture (Fig. 1A). We also measured the systemic appearance of extracellular histones after fracture, because of their cardio-depressive effects in earlier studies^17^. The RIA II animals presented a significant elevation of extracellular histones 6 h after femur fracture compared to baseline (Fig. 1C).

**Figure 2:**
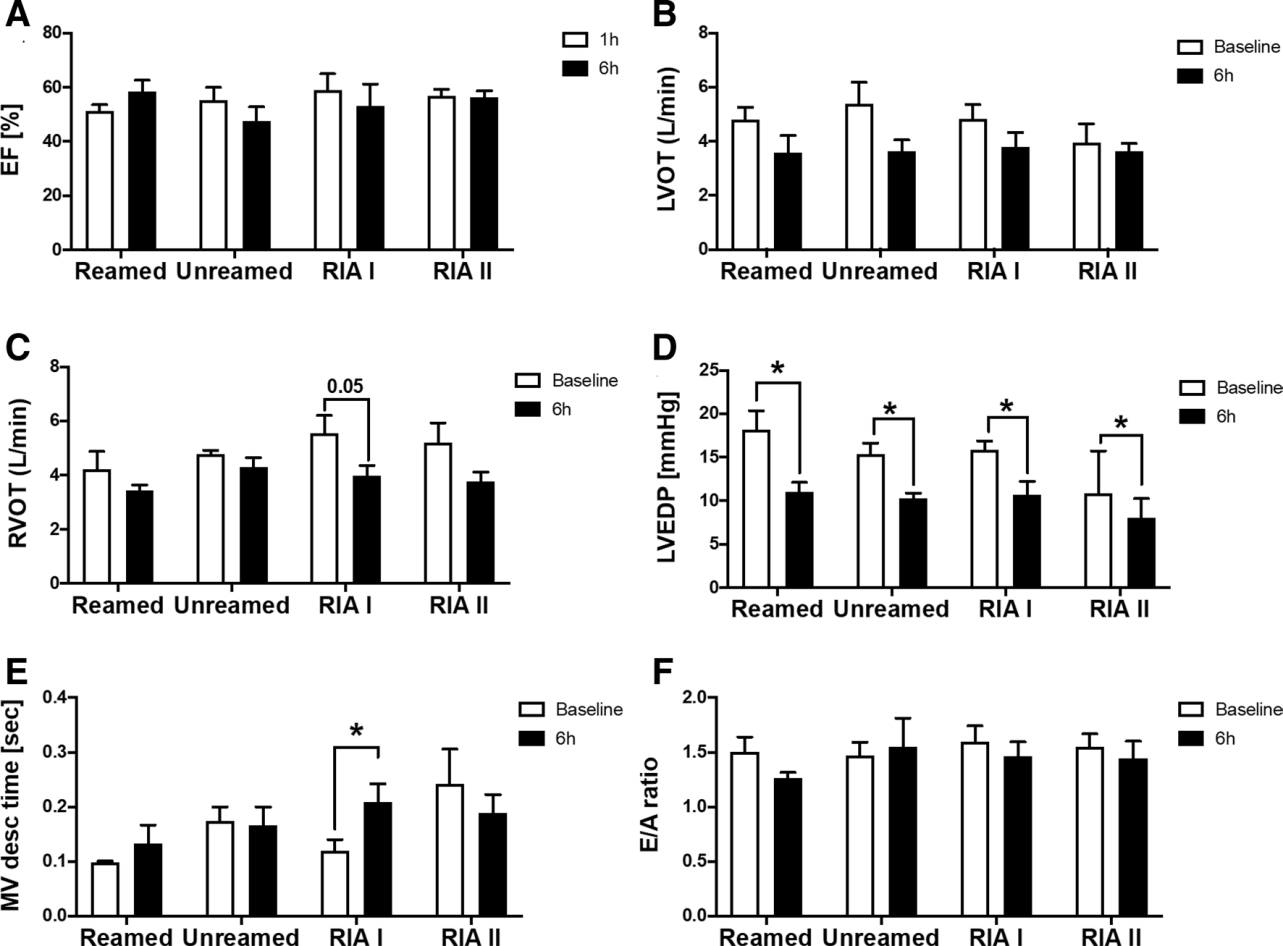
Systolic and Diastolic alterations after fracture: (**A**) Ejection fraction in % measured by Echocardiography at Baseline (white) and 6 h after femur fracture (black) in pigs and either nailing (reamed) reaming (reamed), Reamer-Irrigator-Aspirator 1 (RIA1) or RIA 2. (**B**) Left ventricular outflow tract Cardiac Output in l/min after femur fracture. (**C**) Right ventricular outflow tract cardiac output in l/min. (**D**) Left ventricular end-diastolic pressure (LVEDP) in mmHg bevor and after femur fracture. (**E**) Mitral Valve deceleration time (MV desc time) in sec after femur fracture in pigs compared to baseline. (**F**) Ratio of E to A wave detected amongst the mitral valve (E/A). n = 5 in each group, data were presented as ± SEM, *p ≤ 0.05.

To evaluate the systolic function of the heart, we analyzed the ejection fraction and cardiac output 6 h after fracture. With regard to the ejection fraction, we did not observe any significant changes after fracture compared to baseline measurements (Fig. 2A). The cardiac output presented a by trend reduction of left ventricular outflow tract cardiac output in all fractured animals (Fig. 2B). The right ventricular outflow tract cardiac output showed a significant reduction 6 h after trauma and RIA I treatment (Fig. 2C). In addition to the systolic function, the diastolic function is one important predictor for the outcome of patients. Therefore, we analysed the LVEDP and observed a significant reduction in all animals early after fracture compared to their baseline measurements (Fig. 2D). The MV desc time was also significantly increased after RIA I treatment and subsequently nailing (Fig. 2 E), while the E/A ratio was not altered early after fracture in pigs (Fig. 2F).

**Figure 3:**
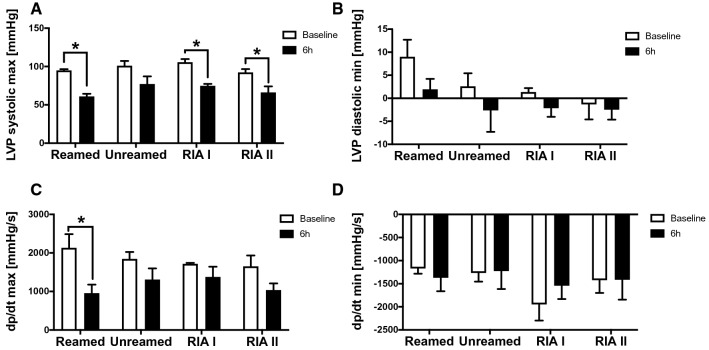
Blood pressure: (**A**) Systolic left ventricular pressure (LVP systolic max) in mmHg measured at BL (white) and 6 h after femur fracture in pigs and either nailing (unreamed) reaming (reamed), Reamer-Irrigator-Aspirator 1 (RIA1) or RIA 2. (**B**) Diastolic left ventricular pressure (LVP diastolic min) in mmHg bevor and after femur fracture. (**C**) dp/dt max measured in mmHg/s. (**D**) dp/dt min in mmHg at baseline and 6 h after femur fracture and treatment. n = 5 in each group, data were presented as ± SEM, *p ≤ 0.05.

Next to the systolic and diastolic function, we also focused on pressure chances in the context of femur fracture and stabilization. We detected a significant reduction of the LVP systolic max pressure early after fracture and reaming, as well as after applying RIA I and II (Fig. 3A). Nevertheless, the LVP diastolic min pressure did not showed any changes in the context of femoral fracture (Fig. 3B). The dp/dt max as an indicator of the cardiac contractility was significantly decreased in animals treated by reaming and intramedullary nailing (Fig. 3C). dp/dt min was not altered after femur fracture (Fig. 3D).

**Figure 4:**
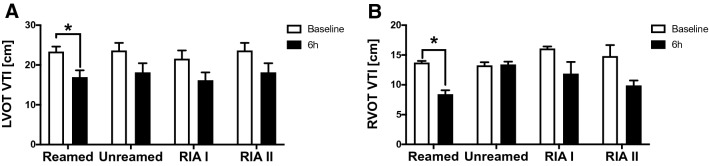
Outflow tract: (**A**) Left ventricular outflow tract velocity time integral (LVOT VTI) in l/min measured by Echocardiography at Baseline (white) and 6 h after femur fracture (black) in pigs and either nailing (unreamed) reaming (reamed), Reamer-Irrigator-Aspirator 1 (RIA1) or RIA 2. (**B**) right ventricular outflow tract velocity time integral (RVOT VTI) in l/min. n = 5 in each group, data were presented as ± SEM, *p ≤ 0.05.

Changes in the outflow tract were previously associated with heart failure. For this reason, we measured the LVOT VTI and observed a significant reduction 6 h after fracture in animals with reaming and subsequently nailing (Fig. 4A). In line with this, the animals treated with reaming showed a reduced RVOT VTI 6 h after fracture compared to their baseline (Fig. 4B).

**Figure 5:**
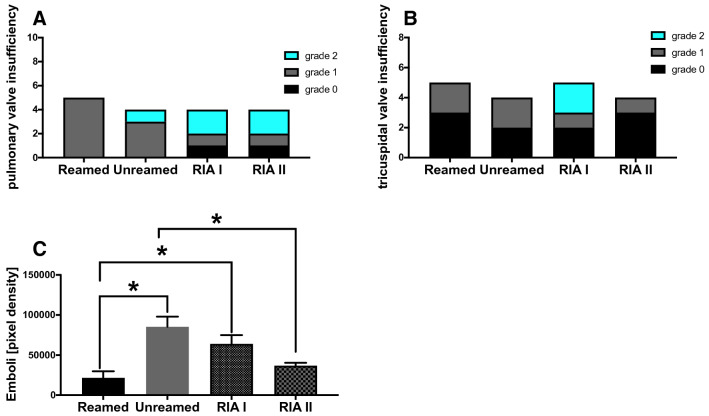
Valvular Insufficiency: (**A**) Number of detected pulmonary valve insufficiency measured by Echocardiography and graded in grade 0 to grade 2 by an experienced investigator 6 h after femur fracture. (**B**) Number of detected tricuspid valve insufficiency. (**C**) Amount of emboli (pixel density) detected in the animals during operative stabilization. n = 5 in each group.

One underestimated entity is the post-traumatic valvular dysfunction. To evaluate the valvular function, we conducted transoesophageal ECHO. We detected a post-traumatic valvular dysfunction of the pulmonary and tricuspid valve after fracture (Fig. 5A,B). Changes in the functionality of the valves could be the result of pulmonary arterial hypertension by fat embolism to the lung. In Fig. 5C we present the amount of emboli detected by echocardiography during unreamed/ reaming or RIA I/II use.

**Figure 6:**
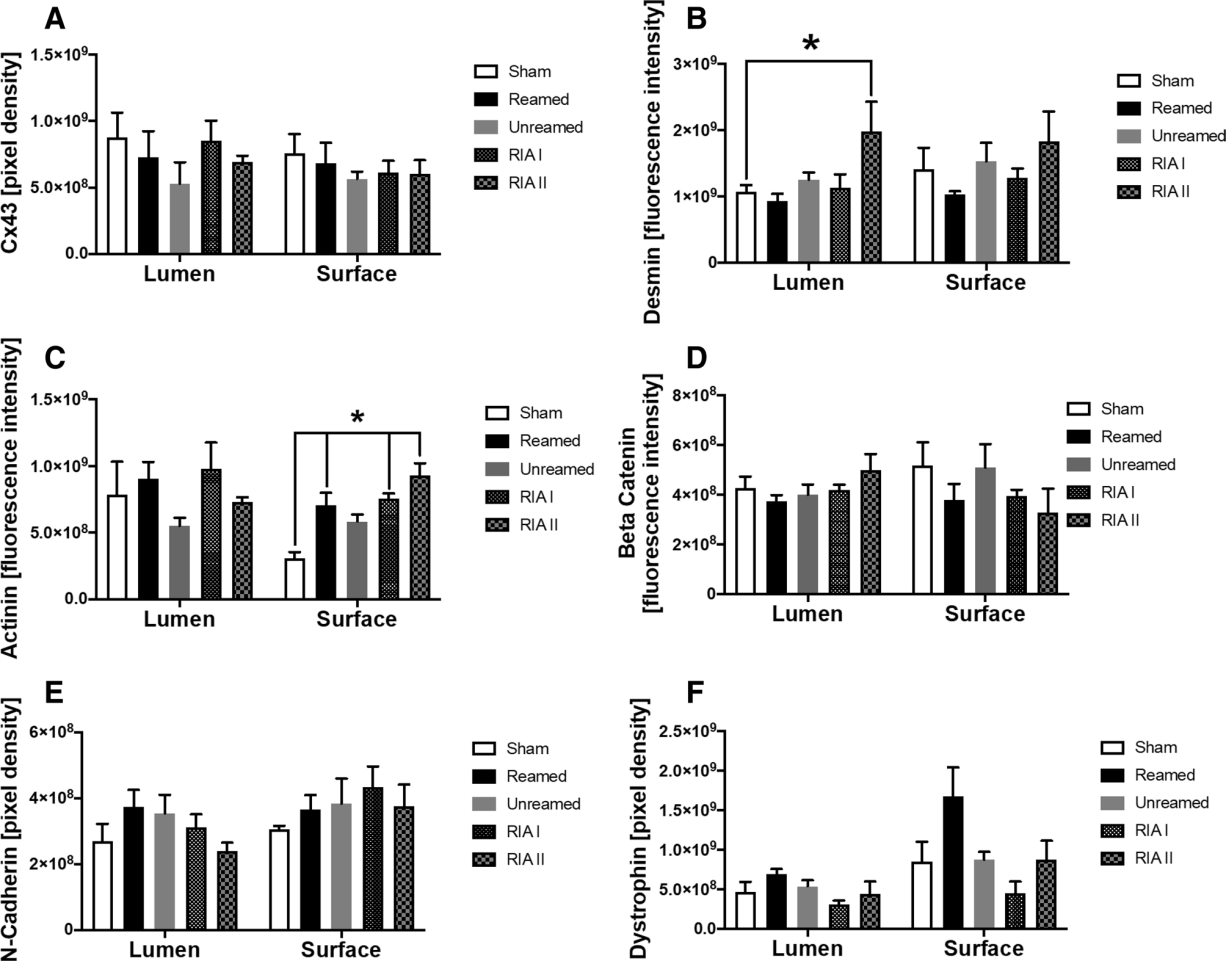
Structural alterations after femur fracture: (**A**) Results of the quantitative analysis of Connexin 43 stained section of the left ventricle 6 h after femur fracture in pigs and either nailing (unreamed) reaming (reamed), Reamer-Irrigator-Aspirator 1 (RIA1) or RIA 2 compared to sham treated animals. (**B**) Results of analysis of immunofluorescence staining of Desmin and (**C**) alpha-Actinin. (**D**) Amount of ß-Catenin measured by immunohistochemical staining of left ventricles after femur fractures. (**E**) Amount of N-Cadherin in the left ventricle of pigs after femur fracture compared to shams. (**F**) Results of quantitative analysis of Dystrophin-staining in left ventricle of fractured pigs. n = 5 in each group, data were presented as ± SEM, *p ≤ 0.05.

Finally, we analysed relevant structural proteins, which are crucial for the cell–cell interaction of cardiomyocytes. We did not encounter changes in the gap junction molecule Cx43 expression after fracture (Fig. 6A). 6 h after trauma and RIA treatment, we observed a significant increase of desmin in the luminal layer of the left ventricle (Fig. 6B). In contrast, all fractured animals presented a local increase of alpha actinin at the superficial layer of the left ventricle compared to sham treated animals (Fig. 6C). Additional analysis of the structural proteins ß-Catenin, N-Cadherin and dystrophin did not show any quantitative alteration early after femur fracture in pigs (Fig. 6D–F).

## Discussion

To the best of our knowledge, the present study showed for the first time a depression of cardiac function after fracture fixation of a unilateral femur fracture. This EMD in the early phase after acute fracture is presented by a systemic increase of cardiac damage indicator proteins, as well as by a functional depression of the heart. In this study, we showed an increase of troponin I and HFABP 6 h after fracture (Fig. 1). In 2000, the European Society of Cardiology and the American College of Cardiology Committee defined an elevation of troponin I or T over the 99th centile of the healthy population as a clinical relevant myocardial damage^18^. In earlier studies, we described a correlation between elevated serum troponin I levels in patients after multiple trauma and the injury severity score (ISS), abbreviated injury scale (AIS) of the chest, the survival and the need for catecholamines^19^. Furthermore, the literature describes well that the systemic elevation of troponin is a sensitive marker for trauma-related complications of the heart. Especially, in combination with functional changes detected by echocardiography, troponin elevation seems to be a diagnostic tool of early myocardial damage^20, 21^. In recently published reports, troponin elevationupon insult has been observed in different experimental trauma models: after multiple trauma in mice^22^, asphyxia and hemorrhage in newborn pigs^23^ and after multiple trauma with hemorrhagic shock in pigs^7^. Unfortunately, all the previously mentioned studies included direct chest trauma models. Therefore, this study is unique because of the detection of cardiac damage with functional alterations in absence of direct mechanical impact on the chest. We further demonstrate the potential origin of the trauma associated troponin in the circulation (Fig. 1D). As the local troponin amount was significantly reduced in the luminal layer of the left ventricle in all fractured animals, it is tempting to hypothesize that this is suggestive of local troponin leakage of cardiomyocytes.

Another biomarker of myocardial damage is the heart fatty acid binding protein (HFABP), which appears earlier in the circulation after myocardial infarction compared to troponin^24^. HFABP is recommended as the most sensitive marker for early cardiac damage. One disadvantage of HFABP is its minimal appearance in skeletal muscle, which is likely damaged in the context of musculoskeletal trauma^25^. A systemic increase of HFABP was recently identified after experimental multiple trauma in pigs^7^. In the present study, all animals demonstrated a significant increase of HFABP early after fracture (Fig. 1A). In case of early myocardial damage, the HFABP seems to be more sensitive at this early trauma phase compared to troponin I. Rising HFABP in the emergency room was previously identified as a reliable predictor of the development of adverse cardiac events^5^.

Figure 1 in this study displays an increase of extracellular histones in the animals with fracture and RIA II reaming. Extracellular histones cause an increase of intracellular calcium in cardiomyocytes via an increase of cell membrane permeability or ROS production^17^. Moreover, histones were recently described to disturb calcium signalling, leading to bradycardia in cardiomyocytes and impair the mitochondrial respiration capacity (HMGB_1/Histonpaper).

As described above, the combination of cardiac damage marker detection and echocardiographic alterations in the function of the heart, were handled as the gold standard in the diagnosis of EMD.

This study is the first to our knowledge that provides a systematic analysis of the cardiac function after long bone fracture, also considering different reaming strategies. First, we analyzed the systolic function of the heart early after femoral fracture in pigs. We found a by-trend reduction of the ejection fraction in all analysed pigs as well as a tendentially reduced LVOT cardiac output (Fig. 2 A/B). The RVOT cardiac output was significantly reduced in animals with fracture and RIA I use (Fig. 2C). In multiple injured pigs, we were able to demonstrate a significant reduction of the ejection fraction and shortening fraction, which was reversible 24 h after polytrauma with haemorrhagic shock^7^.

Diastolic dysfunction can also occur isolated, which is called heart failure with preserved ejection fraction (HFpEF). One diastolic parameter, which is detectable by ECHO and intracardial blood pressure measurements, is the left ventricular end-diastolic pressure (LVEDP). It is considered as an important parameter of ventricular compliance and intravascular volume. LVEDP can identify patients with increased risk of heart failure^26^. This value is elevated (> 15 mmHg) in patients with coronary heart disease or myocardial ischemia^26, 27^. Diastolic function measured by increased LVEDP is a parameter of asynchronous myocardial relaxation and therefore of cardiac stiffness^27^. Our data relating to femur fracture showed a significant reduction of LVEDP in all analyzed animals independent of the stabilization technique (Fig. 2D). A reduced LVEDP after fracture might be the result of a systemic hypovolemia. Uncontrolled bleeding can occur during an operative stabilization of the fracture.

In addition to LVEDP, also the E/A ratio is mentioned as a diastolic parameter^27^. As presented in Fig. 2F, we did not observe any changes in the E/A ratio 6 h after fracture with regard to the different fracture stabilization techniques. In addition, we measured the MV desc time as increases of it can be associated with diastolic dysfunction. In the RIA I group, the animals showed a significant increase of MV desc time (Fig. 2E). This is interesting, because this group also showed a systolic dysfunction measured by RVOT cardiac output. During sepsis, a diastolic dysfunction strongly correlates with adverse clinical outcome as the diastolic compliance partially compensates decreases in systolic contractility^28^.

Beyond the diastolic and systolic function, we analysed alterations in the blood pressure after femur fracture in pigs. All animals of the reaming groups (reamed, RIA I and RIA II) showed a significant reduction of the systolic LVP (Fig. 3A). This could be a consequence of blood loss after femur fracture and operative treatment depending on longer operation time and invasiveness of the treatment. In earlier studies in geriatric patients, no significant differences in blood loss, the need of transfusion or haemoglobin/haematocrit were detected between reamed and unreamed femoral fractures. While a significant shorter duration of operation was needed in the unreamed group, both techniques showed satisfactory results in stabilization^29^. In addition, treatment with RIA I showed a relevant blood loss in 3.22% of patients, which needed transfusion^30^.

In the present study, we detected a reduction of the LVOT VTI and RVOT VTI 6 h after femur fracture and reaming in pigs (Fig. 4A/B). A reduction of the LVOT VTI has been described as a predictor of heart failure and increased mortality^31^. Moreover, the LVOT VTI is a sensitive systolic marker of low cardiac output, cardiogenic shock and impaired ability to keep up the systemic tissue perfusion and metabolic demands^31^.

After car accidents, single case reports of severe valvular regurgitations, rupture of the anterolateral papillary muscle or atypical septum defects were described^32, 33^. In the present study, we newly observed the development of valvular insufficiency in the early phase after femur fracture. Especially the pulmonary and tricuspid valve showed abnormalities after femur fracture, which did not depend on the treatment strategy (Fig. 5 A/B). This is a surprising finding because of the common consensus that traumatic valvular lesions result from high-energetic direct trauma on the chest. Usually, the literature describes the sudden deceleration/compression of the blood column in the heart during the vulnerable phase of the cardiac cycle as the main mechanism causing damage^34^. However, we argue that we should not only consider the mechanical impact on the heart but also the inflammatory component and pulmonary embolism following fracture and fracture stabilization of the valvular insufficiency development. In the context of chest trauma, the pre-existing valvular diseases are associated with a high risk of post-traumatic valvular disorders^35–38^. Furthermore, pulmonary arterial pressure is described to be elevated in case of fracture-induced fat embolism. This observation was independently of the stabilization technique (reamed vs. unreamed) and returned to baseline values 24 h after fracture in canines^39^. In the present study emboli occurred in the right ventricle during fracture stabilization of all types although to various degrees. In earlier experimental studies in pigs fatal pulmonary embolism has been observed after fracture reaming^40^. In a model of intact femur reaming lowering the intramedullary pressure during reaming obtained by RIA reduced bone marrow extravasation^41^.

Pulmonary arterial hypertension is a common reason of valvular insufficiency: 90% of tricuspid regurgitation were caused by pulmonary arterial hypertension^42^. Therefore, further studies are necessary to analyze the development of post-traumatic valvular dysfunction and the inflammatory role as well as the role of pulmonary hypertension in this entity.

Moreover, we detected alterations of cardiomyocytes structural and functional proteins after femur fracture. The important z-disc associated intermediary filament desmin was significantly increased in the left ventricle of pigs with femur fracture and RIA II treatment. This is in line with earlier studies demonstrating an increase of desmin after multiple trauma in pigs^7^. Consequences of desmin alterations are well-described in the context of desminopathies. Therefore, the biomechanics of the heart as well as the calcium balance is influenced in case of desminopathies^43^. The amplitude of calcium handling is affected by desmin. High amounts of desmin may alter the ryanodine receptors function and distribution^43^. Research also links mutations in the desmin gene to cardiomyopathy in up to 50% and to cardiac conduction disease or arrythmia in nearly 60% of patients^44^. A high level of TNF has been shown to induce the cleavage of desmin by caspase-6, which leads to a loss of localization of desmin at the intercalated discs and therefore to an accumulation in CMs^45^. In the present report alterations in the distribution of desmin were absent after femur fracture. 6 h after trauma, desmin was still located at the z-discs. In mice with diastolic dysfunction and in guinea pig with heart failure, an elevation of local desmin in the heart was reported^46, 47^. Both a diastolic dysfunction and an increase of desmin was observed in the RIA II group of the present study. Moreover, Gard et al. (2005) described a remodelling of gap junctions in desmin-related cardiomyopathies^48^. A mislocalization of connexin 43, one of the most important gap junction molecules in the heart, is a common entity after multiple trauma in pigs, chest trauma in rats, multiple trauma in mice and psychosocial stress-related cardiomyopathies in mice^7, 22, 49, 50^. The gap junction profile is crucial for the ion balance in the cardiomyocytes, the development of arrhythmias and therefore responsible for a coordinated contraction as a functional syncytium. Although alterations of connexin 43 have been linked to many entities of ischemic or non-ischemic origin^51–53^, we did not observed a change of the connexin 43 amount after femur fracture (Fig. 6A). Furthermore, we analysed the protein amount of the structural protein alpha actinin early after trauma. As a sign of early myocardial damage after long bone fracture, we observed a significant rise of alpha-actinin in the superficial layer 6 h after trauma in all fractured animals compared to sham treated pigs (Fig. 6). Alpha-actinin is a kind of responder to stretch and mechanical tension and therefore is responsible to the cardiac adaption to hemodynamic changes^54^. A significant increase of alpha-actinin might be a reactive adaption to the traumatic demand on the heart. Earlier studies showed a reduction of alpha-actinin 72 h after multiple trauma in pigs^7^. Alpha actinin is linked to L-type calcium channels^55^. Therefore, changes in the calcium handling of cardiomyocytes might result from the alterations in the structural proteins. Moreover, mutations of the alpha-actinin-2 gene were associated with the development of cardiomyopathy with dilatation of the left ventricle and reduction of the contractility of the whole heart^56, 57^. Sheng et. al (2016) showed an increase of alpha actinin and desmin in case of diastolic dysfunction in mice, while a dislocation was not described^47^. Next to other molecules like catenin and vinculin, alpha actinin builds the connection between actin-filaments and the fasciae adherents build by N-Cadherin. Caused by their important role in the intercalated discs, this study analysed the collective amount of N-Cadherin and beta-Catenin. In the present study, there was no change in the protein amount of N-cadherin after femur fracture. We also did not observe alterations in the amount of ß-catenin. The fact, that we could not observe changes in these proteins, might be caused by the early timepoint. Therefore, further long-time studies are necessary to evaluate the changes of ß-catenin, N-Cadherin and plakoglobin in hearts after fracture and in combination with chest trauma.

## Conclusion

This is the first study to describe the development of functional impairment in the context of Early Myocardial Damage (EMD) after long bone fracture in pigs. In our study, troponin and HFABP were elevated and structural cardiac damage occurred. Alterations in the systolic and diastolic function occurred and were accompanied by emboli during fracture treatment and a pulmonary and tricuspid valvular insufficiency.

## Material and methods

### Porcine femur fracture model

This porcine model follows the framework of the TREAT consortium. The experimental setting is already published^58^. The animal housing and the experimental protocols were approved by the Cantonal Veterinary Department, Zürich, Switzerland (licence number: ZH 138/2017). The experiments were designed in accordance with the Swiss Animal Protection Law and conformed to the European Directive 2010/63/EU of the European Parliament and Council on the Protection of Animals used for scientific purpose and to the Guide for the Care and Use of Laboratory Animals (Institute of Laboratory Animal Resources, National Research Council, National Academy of Sciences, 2011).

In this study we included functional data, blood samples and tissue samples of 25 male pigs (sus scrofa domestica). 20 of these animals underwent a femur fracture. 5 animals underwent the sham procedure, which includes anaesthesia and instrumentation.

After general anaesthesia and baseline measurements, we induced the femoral fracture in 20 animals by using a bolt gun (Blitz-Kernen, turbocut JOBB HGmbH, Germany). The gun was loaded with cattle-killing cartridges (9 × 17, DynamitNobel AG, Troisdorf, Germany). 60 min after trauma, we randomized the femur fracture group in four therapy arms. Pigs received either femoral nailing without reaming (unreamed, n = 5), with reaming and subsequently nailing (reamed, n = 5), with two different Reamer-Irrigator-Aspirator (each n = 5) and subsequently nailing. For reaming a normal drill was utilized to evacuate the intramedullary material (no cooling, higher chance of fat embolism). The RIA system was originally developed to reduce local and systemic complications during intramedullary reaming of long bones. During reaming, continuous irrigation and aspiration of the medullary cavity content reduce the pressure in the medullary cavity and therefore reduce the systemic appearance of bone marrow components. Next to this effect a synchronous cooling lead to reduction of thermic bone damage during reaming^59^. In the RIA I group, the reaming head had holes so that fluid was being irrigated and aspirated continuously (lower pressure = lower risk of iatrogenic shaft fractures, lower embolism rate, less heat, potential removal of infected tissue). The RIA II group only differed from the RIA I because of a reduced diameter, which improved control of irrigation and suction and allowed a more ergonomic interface. As internal stabilization a shortened conventional tibia nail was inserted.

After finishing the fracture stabilization hemodynamic parameters were continuously monitored for 6 h. Serum and plasma samples were collected at baseline and at the end of the observation period after 6 h. The animals were euthanized under deep general anaesthesia by intravenous Na-Pentobarbital overdose. Tissue samples of the left ventricle were obtained 6 h after fracture. For detailed analysis, we separated the superficial and the luminal layer of the ventricle. The tissue was fixed with 4% formalin, followed by embedding it in paraffin.

### Transoesophageal echocardiography (TOE)

To evaluate the cardiac function after femur fractures in pigs, we conducted a transoesophageal echocardiography at baseline and 6 h after trauma. To do so, we used a standard ultrasound machine (Cx50 xMATRIX, Phillips Healthcare, Germany with the × 7-2t probe and the S5-1 ultrasound probe for additional transthoracic measurements). An experienced investigator measured the following echocardiography parameters in the pigs: ejection fraction (EF) calculated as *EF* (%) = (*EDV* − *ESV) 1/EDV* × 100 (EDV = end-diastolic volume; ESV = end-systolic volume), the cardiac output (CO) in l/min, the mitral deceleration time (MX desc time) in s, the ratio (E/A) of peak velocity blood flow in early diastole (the E wave) to the peak velocity flow in late diastole caused by atrial contraction (the A wave). Furthermore, the left ventricular outflow tract velocity time integral (LVOT VTI) in cm and the right ventricular outflow tract velocity time integral (RVOT VTI) were detected. Additionally, the heart valve function was evaluated by the means of colour Doppler echocardiography imaging. The observed insufficiencies were rated from grade 0 (no insufficiency) to grade 3 (massive insufficiency) by an experienced veterinarian. We measured insufficiencies in the pulmonary and tricuspid valve 6 h after trauma.

Moreover, we analysed the continuously measured arterial blood pressure curves and placed a pig tail catheter (5Fr) in the left ventricle of the pigs at baseline and 6 h after fracture^60^. The following parameters were included in this study: the left ventricular end-diastolic pressure (LVEDP) in mmHg, maximal left ventricular/systolic pressure (LVP max) in mmHg, minimal left ventricular/diastolic pressure (LVP min) in mmHg, maximum positive value of the first derivative of the pressure that occurs during cardiac cycle (+ dp/dt max) in mmHg/s, maximum negative value of the first derivative of the pressure that occurs during the cardiac cycle (− dp/dt min) in mmHg/s. During operative stabilization, we further conducted measurements of embolism via echocardiography. Therefore, the number of emboli was detected in echocardiography images while unreamed, reaming or RIA I/II use by using ImageJ software.

Heart-fatty acid binding protein (HFABP), Troponin I and Histone -ELISA in pigs To evaluate the cardiac damage after fracture, we analyzed the systemic release of HFABP and troponin I by using ELISA kits according to the manufacturer’s instructions: HFABP was detected in serum samples by a cardiac fatty acid binding protein ELISA kit (life diagnostics, West Chester, PA, USA). Troponin I was analyzed by an Ultrasensitive Pig Cardiac Troponin-I ELISA Kit (Life Diagnostics, West Chester, PA, USA). Systemic Histones were measured in pig serum by using a cell death detection ELISA kit (Hoffmann-La Roche, Indianapolis, IN, USA). A histone mixture (containing H2, H2A, H2B, H3, H4) (Sigma, St.-Louis, Missouri, USA) was used to establish a standard curve.

### Immunohistochemistry

For immunohistochemistry, the formalin-fixed, superficial and luminal tissue of the left ventricle was de-waxed and hydrated. Furthermore, the unspecific binding sides were blocked with 10% goat Serum. For Connexin (Cx43) staining rabbit anti-pig Cx43 (Cell Signaling Technology, Danvers, MA, USA) primary antibody was used. As a secondary antibody system, we used the Dako REAL Detection System kit (Dako, Glostrup, Denmark) in accordance to the manufactures introductions. To contribute a quantification the signal density was measured in 3 randomly chosen stained slides and layer (Axio ImagerM.1 microscope; Zeiss AxioVision software 4.9 (Zeiss, Jena, Germany). Results are presented as mean density of each group (arbitrary units).

### Immunofluorescence

Tissue of the left ventricle (superficial and luminal layer) was also dewaxed and hydrated. ASs primary antibody, we used a rabbit anti-α-actinin (clone N1N3) (GeneTex, Ivine, CA, USA) and a mouse anti-desmin (GeneTex, Irvine, CA, USA). AS secondary antibody a goat anti-rabbit (AF-488) (Jackson Immuno research Laboratories, West Grove, PA, USA) as well as a goat anti-mouse (AF-647) (Jackson Immuno Research Laboratories, West Grove, PA, USA) antibody was used. We further stained the sections with an anti-Dystrophin (Abcam, Cambridge, UK) antibody, as well as a N-Cadherin-antibody (Abcam, Cambridge, UK). Further, B-Catenin (Abcam, Cambridge, UK) and Troponin I (Abcam, Cambridge, UK) were detected by staining. Counterstaining was conducted with Höchst 33342 (Sigma, Darmstadt, Germany). Following staining, sections were mounted with ProLong Gold Antifade Reagent (Invitrogen, Carlsbad, CA, USA). For quantification, the Axio ImagerM.1 microscope and the Zeiss AxioVision software 4.9 (Zeiss, Jena, Germany) was also used. Results are presented as mean density of each group (arbitrary units).

### Statistical procedures

Data were analyzed by 2-way ANOVA followed by Sidak’s multiple comparison test. Changes were considered statistically significant at the threshold of p ≤ 0.05. Statistical analysis and graphical presentation were conducted using the GraphPad Prism 7.0 software (GraphPad Software, Incorporated, San Diego, Ca, USA). The values are presented as mean ± SEM in all graphs.
